# Fermented garlic as a functional food strategy for malnutrition: microbial ecology, bioactive compounds, and clinical perspectives

**DOI:** 10.3389/fnut.2026.1839155

**Published:** 2026-06-11

**Authors:** Miracle Amarachukwu Emmanuel-Fashagba, Yemisi Dorcas Obafemi, Solomon Uche Oranusi

**Affiliations:** 1 Department of Biological Sciences, College of Science and Technology, Covenant University, Ota, Ogun State, Nigeria; 2 Food and Feed Immunology Group, Laboratory of Animal Food Function, Graduate School of Agricultural Science, Tohoku University, Sendai, Japan

**Keywords:** bioactive compounds, fermented garlic, functional foods, gut microbiota, lactic acid bacteria, malnutrition

## Abstract

Fermented garlic (*Allium sativum*) represents a promising functional food with potential applications as a complementary nutritional intervention for malnourished populations. Through microbial fermentation and thermal processing two mechanistically distinct pathways, garlic undergoes significant biochemical transformations that enhance the availability of bioactive compounds, including S-allyl-L-cysteine (SAC), polyphenols, and *γ*-aminobutyric acid (GABA), which collectively contribute to improved antioxidant capacity and gut health. This comprehensive review examines the microbial ecology underlying garlic fermentation, the biochemical pathways that generate bioactive metabolites, and the mechanistic basis by which fermented garlic employed in the broader food fortification strategy or incorporated into fortified therapeutic food formulations targeting clinical malnutrition, may support nutritional recovery in the context of Environmental Enteric Dysfunction (EED), the dominant gut pathology underlying stunting and wasting in low- and middle-income countries (LMICs). Fermented garlic is a bioactive-dense nutritional adjuvant rather than a macronutrient source, its clinical relevance lies in potential enhancement of gut barrier integrity, reduction of mucosal inflammation, and support of micronutrient bioavailability, rather than direct caloric contribution. Preclinical evidence from animal models demonstrates improvements in intestinal morphology, metabolic parameters, and immune function, suggesting potential utility in nutritionally stressed populations. However, well-designed human clinical trials specifically examining fermented garlic in malnourished populations are currently underrepresented in the literature, and all translational implications discussed herein remain preliminary. Substantial research gaps persist regarding optimal dosage, long-term clinical safety, and standardization of fermentation protocols. This review identifies critical research priorities necessary to establish fermented garlic as a scalable, culturally acceptable food-based complementary intervention for vulnerable populations worldwide.

## 1 Introduction

Malnutrition remains one of the most pressing challenges facing global public health, particularly in low- and middle-income countries (LMICs) across Africa, South Asia, and Southeast Asia. The condition encompasses a spectrum of nutritional disorders, including protein-energy malnutrition (PEM), micronutrient deficiencies, and overnutrition, all of which have profound consequences for physical development, immune function, and long-term health outcomes (1). The World Health Organization estimates that approximately 735 million people globally experience chronic hunger, with malnutrition contributing directly to nearly 45% of deaths in children under 5 years of age, primarily through increased susceptibility to infectious diseases (2).

Traditional nutritional interventions including dietary diversification, food fortification, and supplementation programs have demonstrated efficacy in enhancing nutritional outcomes. However, implementation in resource-limited settings remains constrained by economic cost, accessibility challenges, poor cultural adherence, and limited sustainability beyond externally funded programs (2). Consequently, growing recognition exists among nutrition researchers and public health practitioners that innovative, locally derived food-based interventions offer promise for addressing malnutrition in vulnerable populations.

A critical but underappreciated dimension of childhood malnutrition in LMICs is Environmental Enteric Dysfunction (EED) a subclinical condition characterized by chronic intestinal inflammation, villous blunting, increased intestinal permeability (“leaky gut”), microbial translocation, and dysbiosis (3). EED perpetuates malnutrition by impairing the absorptive capacity of therapeutic foods, even when adequate nutrition is provided. Interventions capable of repairing gut barrier integrity and modulating intestinal microbiota therefore represent a high-priority strategy in nutritional rehabilitation programs.

Fermented foods have occupied a central role in human dietary traditions for millennia, serving not only as preservation mechanisms but as nutritional enhancers (4). Contemporary research has demonstrated that fermentation processes substantially alter the biochemical composition of food substrates, improving nutrient bioavailability, reducing anti-nutritional compounds, and introducing beneficial microorganisms that support gut health and immune function (5). Across African communities, traditional fermented foods including Ogi (fermented maize porridge), Kunu-zaki (fermented cereal beverage), and Dawadawa (fermented locust bean condiment) have been documented to enhance dietary quality and contribute to mitigation of micronutrient deficiencies (6).

Garlic (*Allium sativum*) is globally recognized for its antimicrobial, antioxidant, and immunomodulatory properties. However, the bioavailability of garlic’s bioactive compounds is limited when consumed raw, and its characteristic pungent odor limits palatability and dietary acceptability (7). Fermentation emerged as a strategic processing approach to enhance garlic’s functional properties, enhance palatability, and generate stable bioactive compounds with demonstrated health-promoting potential (8). Two primary fermented garlic products have received considerable scientific attention: lactic acid-fermented garlic, produced through microbial fermentation by lactic acid bacteria (LAB), and black garlic, generated through controlled thermal aging at elevated temperature and humidity (9). These two pathways are mechanistically distinct and must not be conflated: lactic acid fermentation is driven by microbial enzymatic catalysis, while thermal aging is a heat-driven chemical process occurring in the absence of active microbial metabolism.

Importantly, fermented garlic is not positioned as a primary macronutrient source. Its potential contribution to addressing malnutrition lies in its role as a bioactive-dense complementary nutritional intervention specifically, one that may enhance gut barrier function, reduce intestinal inflammation, support micronutrient bioavailability, and improve the efficacy of existing therapeutic food programs (e.g., ready-to-use therapeutic foods, RUTF) in populations affected by EED.

The present review synthesizes current understanding of garlic fermentation processes, the microbial ecology driving biochemical transformations, the bioactive compounds generated during fermentation, and the mechanistic pathways through which fermented garlic may influence nutritional status and immune function in the context of malnutrition. By integrating preclinical evidence, available observational human data, and identified research gaps, this review aims to clarify the potential of fermented garlic as a scalable, culturally acceptable complementary functional food strategy for addressing malnutrition in vulnerable populations, with particular focus on LMICs including sub-Saharan Africa.

### 1.1 Review methodology

Studies were included if they: (1) were peer-reviewed original research articles, meta-analyses or systematic reviewsn published in English; (2) examined LAB microbial ecology, garlic fermentation processes, bioactive compound characterization, or nutritional and health outcomes of fermented or thermally aged garlic; and (3) were published between January 2010 and March 2026, with key foundational pre-2010 studies included where mechanistically essential. Studies were excluded if they analyzed dried or raw garlic without fermentation or thermal processing, were conference abstracts, or were not peer-reviewed. A PRISMA-style flow diagram summarizing study selection is provided as Supplementary Figure S1.

Search terms included: “fermented garlic,” “*Allium sativum* fermentation,” “lactic acid bacteria garlic,” “black garlic bioactive,” “S-allyl-L-cysteine,” “garlic gut health,” “garlic malnutrition,” “garlic Environmental Enteric Dysfunction,” “garlic GABA,” and “garlic clinical trial.” Boolean operators (AND, OR) combined terms. Reference lists of retrieved articles were hand-searched for additional eligible sources.

A systematic literature search was conducted across four electronic databases: PubMed/MEDLINE, Scopus, Google Scholar and Web of Science. The search covered publications from January 2010 to March 2026. Searches were conducted independently by two authors (MAE-F and YDO), with discrepancies resolved by consensus with the supervising author (SUO).

Given that this review integrates evidence across food science, clinical nutrition and microbiology, a transparent description of the literature search strategy is provided to enable assessment of coverage completeness and reliability, consistent with Frontiers journal standards for narrative reviews.

## 2 Microbial fermentation of garlic

### 2.1 Fermentation pathways: lactic acid fermentation and thermal aging

**Figure 1:**
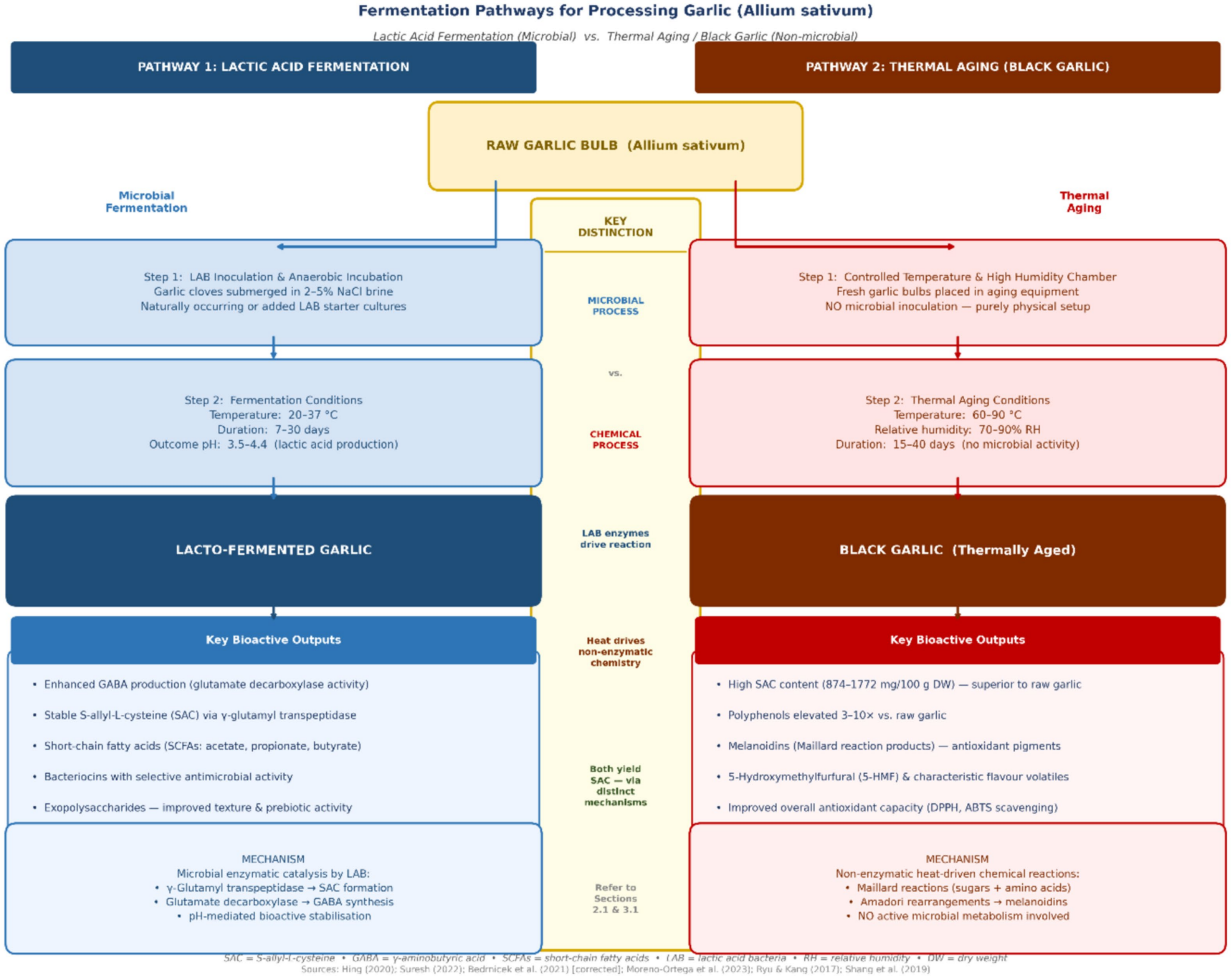
Fermentation pathways for processing garlic (*Allium sativum*). The two pathways are mechanistically distinct: lactic acid fermentation is driven by microbial enzymatic catalysis, while thermal aging (black garlic production) is a heat-driven chemical transformation occurring in the absence of active microbial metabolism. Original author-created schematic. Data sources cited in figure legend (7, 8, 10–13).

Garlic fermentation proceeds through two distinct pathways, each characterized by unique biochemical mechanisms, microbial involvement, and resulting bioactive profiles. Lactic acid fermentation is a microbially mediated process driven by enzymatic catalysis, whereas thermal aging (black garlic production) is a heat-driven chemical transformation occurring in the complete absence of active microbial metabolism. Understanding these pathways and distinctions is essential for optimizing fermentation protocols, predicting and interpreting functional outcomes and avoiding conflation of their mechanistic bases (as highlighted in Figure 1).

#### 2.1.1 Lactic acid fermentation

In lactic acid fermentation, peeled garlic cloves are submerged in brine solutions containing 2–5% sodium chloride and incubated at temperatures ranging from 20 to 37 °C for 7–30 days (10). During fermentation, naturally occurring or inoculated lactic acid bacteria metabolize available carbohydrates, producing organic acids predominantly lactic acid which lower the pH to approximately 3.5–4.4 (11). This acidification inhibits spoilage microorganisms, facilitates enzymatic conversion of sulfur-containing compounds via microbial *γ*-glutamyl transpeptidase and glutamate decarboxylase activity, and creates conditions favoring synthesis of bioactive metabolites. Lacto-fermented garlic exhibits reduced pungency, enhanced digestibility, and increased concentrations of GABA and SAC.

#### 2.1.2 Black garlic production (thermal aging)

In contrast, black garlic production relies on prolonged thermal aging rather than active microbial fermentation (Figure 1). Fresh garlic bulbs are subjected to controlled conditions of approximately 60–90 °C temperature and 70–90% relative humidity for 15–40 days (12). During this process, non-enzymatic browning reactions particularly Maillard reactions between reducing sugars and amino acids, and Amadori rearrangements drive the transformation of garlic tissues in the absence of microbial activity. These thermal reactions generate dark pigments (melanoidins), characteristic flavor compounds, and substantially increased concentrations of SAC and polyphenols (13). The mechanisms of SAC formation in lactic fermentation (enzyme-mediated deglutamylation by microbial *γ*-glutamyl transpeptidase) are therefore mechanistically distinct from those in thermal aging (non-enzymatic heat-driven conversion), though both pathways ultimately yield this key bioactive compound.

### 2.2 Microbial ecology of lactic-fermented garlic

**Figure 2:**
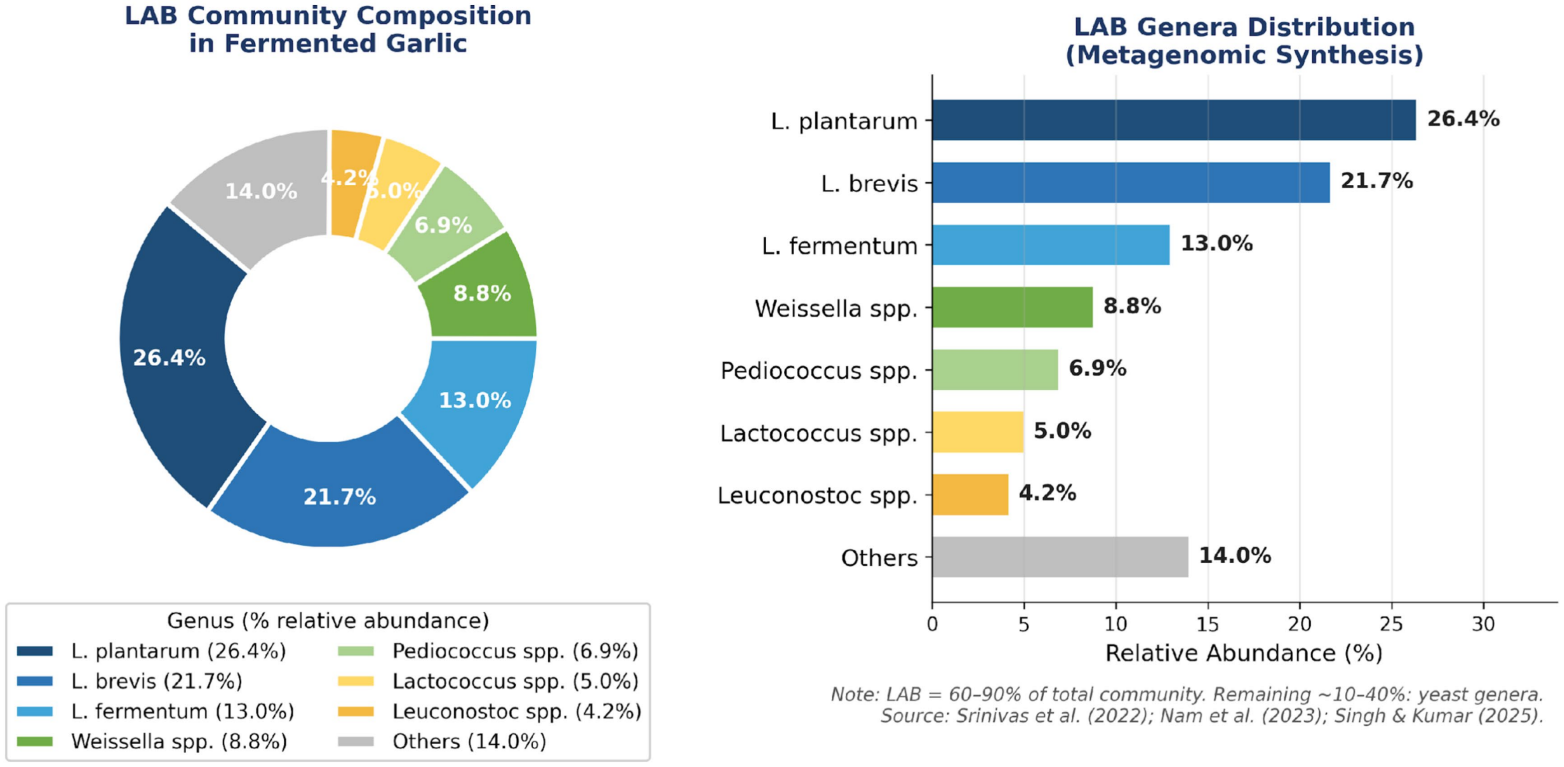
Relative abundance of dominant lactic acid bacteria (LAB) genera in fermented garlic based on metagenomic data synthesis. Left: Proportional donut chart. Right: Horizontal bar chart with percentage annotations. LAB constitute 60–90% of the total microbial population; remaining community is predominantly composed of yeast genera. Original author-created data visualisation. Data synthesized from Srinivas et al. (14), Nam et al. (19), and Singh and Kumar (23).

The microbial communities that develop during garlic fermentation are characterized by complex ecological succession, with lactic acid bacteria (LAB) emerging as the dominant microorganisms responsible for fermentation and bioactive metabolite production (Figure 2). Culture-based studies combined with metagenomic sequencing have consistently identified LAB as the principal microbial group driving fermentation, typically constituting 60–90% of the total microbial population (14, 15).

#### 2.2.1 Dominant LAB species

Among LAB commonly detected in fermented garlic, *Lactiplantibacillus plantarum* and *Levilactobacillus brevis* (formerly *Lactobacillus brevis*) are frequently dominant species. These bacteria are metabolically well-adapted to acidic, low-oxygen environments characteristic of fermented foods, and contribute to rapid acidification through efficient production of lactic acid and other organic acids (16). Beyond acidification, the metabolic activity of these dominant LAB species leads to synthesis of functional metabolites including antimicrobial compounds (bacteriocins), exopolysaccharides, and *γ*-aminobutyric acid (GABA), which collectively improve the nutritional and functional properties of fermented garlic (17, 18).

Quantitative metagenomic evidence supports the dominant status of these species: *Lactiplantibacillus plantarum* accounts for approximately 26.4% and *Levilactobacillus brevis* for approximately 21.7% of the total LAB community in fermented garlic systems, together comprising nearly half the LAB population across multiple independent studies (14, 19). The term “frequently” is therefore supported by quantitative prevalence data rather than qualitative observation alone.

#### 2.2.2 Secondary LAB genera

Other LAB genera including *Leuconostoc, Pediococcus*, and *Weissella* frequently participate in the fermentation process, typically dominating early fermentation stages where they initiate carbohydrate metabolism, produce organic acids and establish conditions favorable for subsequent proliferation of more acid-tolerant species (20). Their metabolic contributions extend to microbial stability, flavor development and formation of additional bioactive compounds.

#### 2.2.3 Yeast participation

Yeasts including *Saccharomyces cerevisiae*, Candida species, and Pichia species frequently coexist with LAB in fermented garlic ecosystems. These microorganisms contribute through carbohydrate metabolism, production of volatile aroma compounds and synergistic interactions with bacterial populations (21). In mixed fermentations, yeasts may interact synergistically with LAB to enhance metabolite diversity and antioxidant potential (22).

The composition and temporal succession of these microbial communities are substantially influenced by salt concentration, garlic cultivar, fermentation temperature and initial microbial load present on raw garlic (19, 23).

### 2.3 Biochemical transformations during garlic fermentation

Microbial activity during fermentation drives extensive biochemical transformations that fundamentally alter the chemical composition and functional properties of garlic. These changes are primarily driven by enzymatic reactions catalyzed by microbial enzymes and by direct microbial metabolism, which collectively convert precursor molecules into bioactive compounds with substantial nutritional and therapeutic potential.

#### 2.3.1 Organosulfur compound transformation

One of the most significant biochemical transformations involves modification of garlic’s characteristic organosulfur compounds. Enzymatic reactions catalyzed by microbial *γ*-glutamyl transpeptidase facilitate conversion of sulfur-containing precursors into stable compounds including S-allyl-L-cysteine (SAC) and related derivatives (24, 25). These compounds exhibit strong antioxidant and anti-inflammatory properties that contribute substantially to the improved functional value of fermented garlic.

#### 2.3.2 GABA synthesis

Microbial fermentation promotes synthesis of γ-aminobutyric acid (GABA) through decarboxylation of glutamic acid, catalyzed by bacterial glutamate decarboxylase enzymes present in LAB. GABA is an important bioactive compound associated with neurological regulation, stress reduction, cardiovascular health and potential cardiometabolic benefits (18, 26).

#### 2.3.3 Additional metabolite production

Fermentation also produces organic acids, bacteriocins with antimicrobial activity and exopolysaccharides that enhance texture and improve nutrient bioavailability (27, 28). Collectively, these biochemical changes improve the nutritional value, functional properties and sensory characteristics of fermented garlic.

#### 2.3.4 Thermal aging reactions

In black garlic production, biochemical transformations occur exclusively through non-enzymatic Maillard reactions between amino acids and reducing sugars a process mechanistically distinct from microbial fermentation. These reactions generate dark pigments (melanoidins), flavor compounds and increased concentrations of phenolic compounds and antioxidant metabolites (13).

## 3 Bioactive compound profiles and nutritional enhancement

### 3.1 Organosulfur compounds: stability and antioxidant capacity

Fresh garlic contains high concentrations of alliin (S-allyl-L-cysteine sulfoxide), which is rapidly converted to allicin upon tissue disruption. Although allicin exhibits strong antimicrobial and antioxidant activity, it is highly unstable and quickly decomposes into lipid-soluble sulfur compounds such as diallyl disulfide and diallyl trisulfide (8). This instability significantly limits systemic bioavailability and biological efficacy.

**Figure 3:**
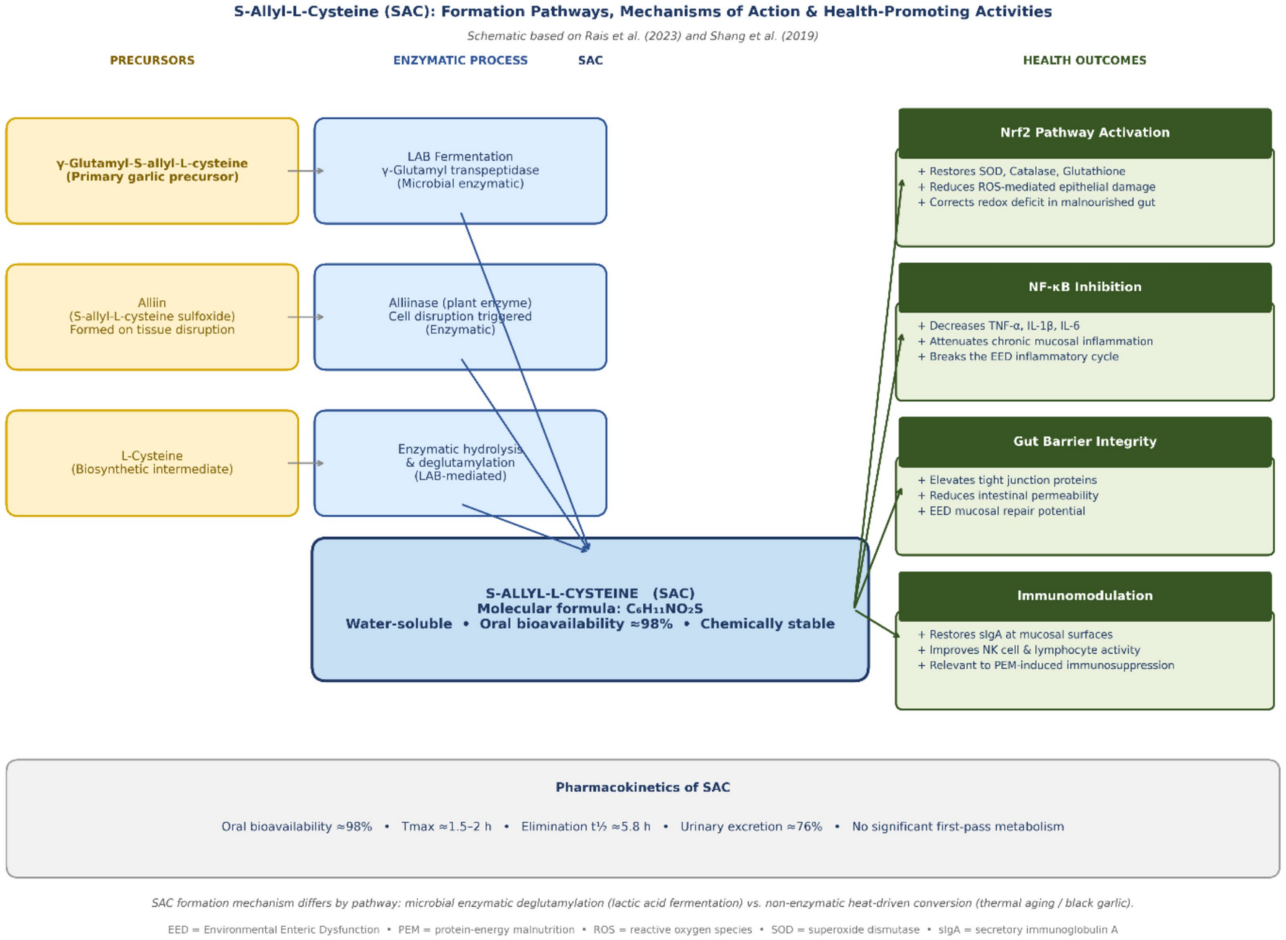
S-Allyl-L-cysteine (SAC): formation pathways, mechanisms of action, pharmacokinetics, and health-promoting activities. SAC arises via enzymatic deglutamylation in lactic acid fermentation and via non-enzymatic heat-driven conversion in thermal aging two distinct mechanistic routes. Schematic based on Rais et al. (41) and Shang et al. (8). Nrf2, nuclear factor erythroid 2-related factor 2; NF-κB, nuclear factor kappa-light-chain-enhancer of activated B cells; GSH, glutathione; SOD, superoxide dismutase; sIgA, secretory immunoglobulin A. Original author-created schematic, based conceptually on Rais et al. (41) and Shang et al. (8).

Fermentation and aging processes substantially shift this profile toward more stable, water-soluble compounds, particularly S-allyl-L-cysteine (SAC). SAC concentrations increase significantly during black garlic production and are associated with improved antioxidant capacity and superior bioavailability compared to allicin (7, 29). SAC has been demonstrated to support endogenous antioxidant systems and regulate inflammatory signaling pathways (Figure 3). Because oxidative stress is frequently elevated in malnourished individuals contributing to immune dysfunction and impaired tissue repair the presence of stable antioxidants such as SAC may help support recovery and restore redox balance (30).

### 3.2 *γ*-Aminobutyric acid and amino acid metabolites

Lactic fermentation can significantly increase γ-aminobutyric acid (GABA) concentration through microbial glutamate decarboxylase activity in lactic acid bacteria (4, 30). Fermented garlic products frequently contain substantially higher GABA levels compared with raw garlic, representing a functionally significant enhancement. Beyond its well-characterized role as a neurotransmitter in the central nervous system, GABA has been associated with metabolic regulation, including effects on blood pressure control, modulation of stress responses and improvement of insulin signaling (29). This metabolic enrichment may contribute substantially to the physiological benefits of fermented garlic consumption.

### 3.3 Polyphenols and Maillard reaction products

**Figure 4:**
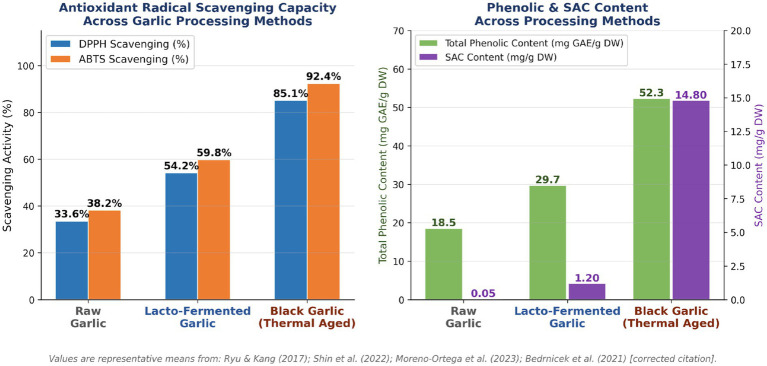
Antioxidant capacity and bioactive content across garlic processing methods. Left: DPPH and ABTS radical scavenging activity (%). Right: Total phenolic content (mg GAE/g DW) and SAC content (mg/g DW). Values are representative means synthesized from cited primary sources (7, 12, 13, 29). Raw garlic values are used as baseline reference. Original author-created data visualisation. Values are representative means from Ryu and Kang (7), Bedrníček et al. (12), Moreno-Ortega et al. (13) and Shin et al. (29).

Thermal aging during black garlic production promotes Maillard reactions between reducing sugars and amino acids, generating melanoidins and enhancing the availability of polyphenolic compounds. Studies have documented substantial increases in total phenolic content and overall antioxidant capacity following garlic aging (7, 29). Polyphenols and Maillard-derived compounds contribute to free radical scavenging, metal ion chelation, and modulation of inflammatory signaling pathways (Figure 4). These antioxidant effects may be particularly beneficial in conditions of nutritional stress, where oxidative damage and pro-inflammatory states frequently contribute to immune dysfunction and impaired nutrient utilization.

A critical observation from comparative analysis of the cited literature is the substantial quantitative variability in bioactive compound concentrations across studies. SAC content ranges from approximately 0.05 mg/g dry weight (DW) in raw garlic to 1.2 mg/g DW in lacto-fermented products and up to 14.8 mg/g DW in optimally aged black garlic, a nearly 300-fold range. Total phenolic content varies from 18.5 to 52.3 mg gallic acid equivalents (GAE)/g DW across processing methods. This heterogeneity is attributable to geographic origin and garlic cultivar, fermentation temperature and duration, LAB strain identity and inoculum density, salt concentration and post-fermentation storage conditions (13, 18). This variability has direct implications for cross-study comparisons and underscores the need for standardized fermentation protocols before clinical translation can be meaningfully pursued.

### 3.4 Micronutrient enhancement and vitamin production

Certain microorganisms involved in vegetable fermentation can synthesize B-group vitamins, including riboflavin and folate (4, 5). Although garlic is not inherently a major source of B vitamins, fermentation may enhance micronutrient availability through two mechanisms: (1) microbial biosynthesis of vitamins, and (2) improved extractability through reduced pH and increased solubility. Additionally, organic acid production during fermentation lowers pH and improves mineral solubility, potentially increasing the bioavailability of iron and zinc (4). These micronutrients are critically deficient in malnourished populations, iron deficiency anemia and zinc deficiency are among the most prevalent micronutrient disorders globally, suggesting that fermented garlic could make a modest but meaningful contribution to addressing micronutrient gaps, particularly when incorporated into diversified dietary strategies in LMICs.

### 3.5 Prebiotic components and short-chain fatty acid production

Garlic naturally contains fructooligosaccharides (FOS) and inulin-type fructans that function as prebiotic substrates. During intestinal passage and fermentation by the colonic microbiota, these carbohydrates are metabolized to produce short-chain fatty acids (SCFAs) including acetate, propionate, and butyrate (31). SCFAs play essential roles in maintaining intestinal barrier integrity, regulating immune responses, and supporting metabolic homeostasis. By providing fermentable substrates and modulating microbial composition, fermented garlic may indirectly promote SCFA production and support overall gut health a mechanism particularly relevant in malnourished populations where gut barrier dysfunction and dysbiosis contribute to continued nutrient malabsorption and perpetuation of the EED cycle.

## 4 Mechanistic basis for nutritional benefits in malnourished hosts

**Figure 5:**
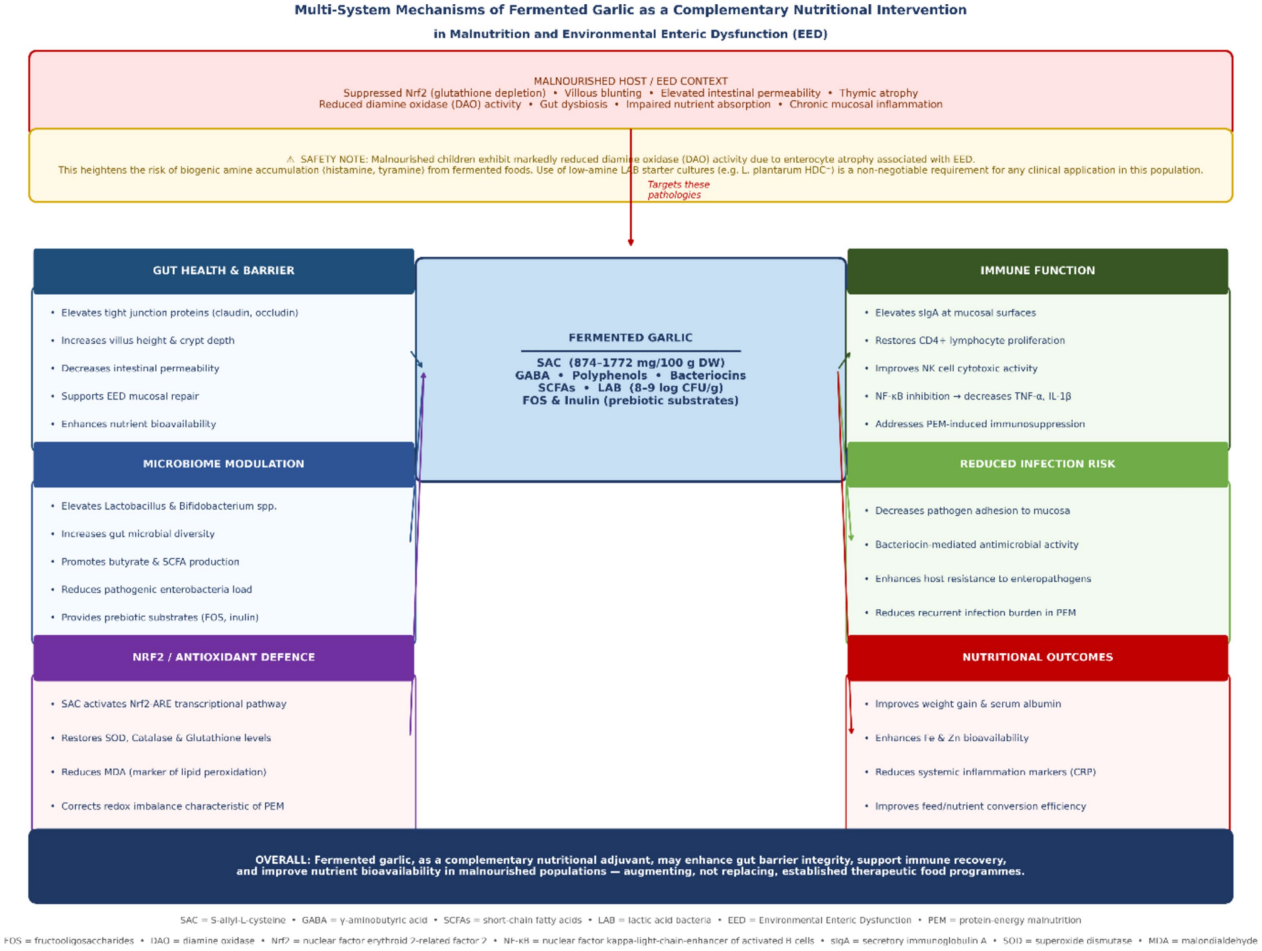
Multi-system mechanisms of fermented garlic as a complementary nutritional intervention in malnutrition and environmental enteric dysfunction (EED). All mechanistic pathways are contextualized for the physiology of the malnourished host. The central box illustrates the bioactive constituents of fermented garlic; surrounding panels describe their mechanistic targets and expected effects. The safety warning highlights the biogenic amine risk specific to malnourished children. SAC, S-allyl-L-cysteine; GABA, gamma-aminobutyric acid; SCFAs, short-chain fatty acids; EED, environmental enteric dysfunction; PEM, protein-energy malnutrition; DAO, diamine oxidase; FOS, fructooligosaccharides.

The potential nutritional benefits of fermented garlic arise from interactions between its bioactive compounds and multiple physiological systems involved in metabolic and immune regulation. Critically, the mechanistic pathways discussed in this section are contextualized specifically for the malnourished host, in whom these pathways are distinctly and often severely dysregulated (Figure 5). Evidence suggests that fermented garlic influences oxidative stress pathways, inflammatory signaling, and gut microbial ecology all of which are closely linked to malnutrition and its sequelae (18, 32).

It is important to distinguish the pathophysiological contexts relevant to this review. In protein-energy malnutrition (PEM) and Environmental Enteric Dysfunction (EED), the Nrf2, NF-κB, and gut barrier pathways discussed below are dysregulated through mechanisms distinct from those observed in metabolic disease states such as obesity or dyslipidemia. In PEM/EED, pathway dysregulation is driven by enterocyte atrophy, glutathione depletion and systemic protein deficit not by caloric excess or insulin resistance. The mechanistic discussion in Sections 4.1–4.3 explicitly addresses each population context and does not conflate malnutrition with metabolic disease.

### 4.1 Modulation of oxidative stress in the malnourished host

Oxidative stress is a common and severe feature of protein-energy malnutrition. In the malnourished host, Nrf2 (nuclear factor erythroid 2-related factor 2) pathway activity is typically suppressed due to glutathione depletion and reduced availability of cysteine precursors the critical substrate for endogenous antioxidant synthesis. This results in reduced activity of antioxidant enzymes including superoxide dismutase (SOD) and catalase, leading to cellular damage, impaired tissue repair, and immune dysfunction.

Fermented garlic exhibits significantly greater antioxidant activity than raw garlic due to increased concentrations of stable organosulfur compounds and polyphenols (7, 29). A key mechanistic pathway involves activation of the Nrf2 signaling pathway by SAC and related compounds (8). SAC, as a bioavailable cysteine-containing organosulfur compound, may directly support Nrf2 reactivation in the malnourished host by providing cysteine substrate for glutathione regeneration. Experimental studies have demonstrated that fermented garlic supplementation reduces lipid peroxidation markers and improves antioxidant enzyme activity, suggesting that its bioactive compounds may support restoration of antioxidant capacity in nutritionally compromised individuals (30).

### 4.2 Anti-inflammatory and immunomodulatory effects in malnourished populations

Malnutrition-induced immunosuppression is characterized by thymic atrophy, impaired T-lymphocyte proliferation, reduced secretory IgA (sIgA) production, and blunted natural killer (NK) cell activity collectively rendering affected individuals highly susceptible to enteropathogens. Chronic inflammation further impairs nutrient metabolism; elevated pro-inflammatory cytokines (TNF-*α*, IL-6) are consistently observed in PEM and EED, creating a vicious cycle of immune activation and continued nutrient depletion.

Bioactive compounds in fermented garlic have been shown to inhibit NF-κB activation, a key transcription factor regulating inflammatory responses (8, 29). Suppression of NF-κB signaling reduces expression of TNF-α and IL-1β. Importantly, NF-κB inhibitory effects and LAB-mediated immunomodulation may be particularly relevant to restoring mucosal immune competence in malnourished populations, where the mucosal immune system is characterized by paradoxical chronic activation alongside suppressed adaptive immunity. Lactic acid bacteria associated with fermentation can also enhance mucosal immunity and stimulate sIgA production (5) an effect of particular importance in populations where mucosal immunity is compromised.

### 4.3 Gut microbiota, intestinal barrier function, and environmental enteric dysfunction

The intestinal microbiota plays a central role in nutrient absorption, immune regulation, and metabolic homeostasis. In malnutrition and EED, dysbiosis is characterized by reduced microbial diversity, depletion of beneficial commensal bacteria (Lactobacillus, Bifidobacterium spp.), and expansion of Gram-negative pathobionts contributing to increased lipopolysaccharide (LPS) translocation, chronic immune activation, and further deterioration of absorptive capacity.

The pathological hallmarks of EED villous blunting, increased intestinal permeability, chronic mucosal inflammation, and microbial translocation represent precisely the physiological targets for fermented garlic’s bioactive mechanisms. Fermented garlic contains prebiotic carbohydrates (FOS, inulin-type fructans) that support growth of beneficial gut bacteria and promote SCFA production (31). Butyrate, the principal colonocyte energy substrate produced by microbial fermentation, directly supports tight junction protein expression (claudin, occludin) and epithelial repair key mechanisms for restoring intestinal barrier integrity disrupted in EED.

The Nrf2-mediated antioxidant pathway, activated by SAC and polyphenols in fermented garlic, may provide additional support for gut mucosal repair through reduction of reactive oxygen species (ROS)-mediated epithelial damage in the inflamed malnourished gut. Collectively, these interconnected mechanisms position fermented garlic as a complementary adjuvant with potential to enhance the efficacy of therapeutic food programs by enhancing intestinal absorptive capacity not by replacing therapeutic foods, but by addressing the gut pathophysiology that limits their effectiveness in EED-affected populations.

## 5 Evidence from preclinical and human studies

### 5.1 Protein-energy malnutrition and nutritional efficiency models

Preclinical studies provide important insights into the potential nutritional and physiological benefits of fermented garlic, particularly in the context of metabolic dysfunction and nutritional stress (Table 1). Experimental models have demonstrated that fermentation enhances the bioavailability of garlic bioactives including SAC, polyphenols, and antioxidant compounds, thereby improving metabolic regulation, immune responses, and gut health (8, 33).

**Table 1:** Preclinical studies of fermented garlic across metabolic and nutritional models.

Metabolic focus	Model/nutritional status	Key findings and mechanisms	References
Obesity and weight management	HFD rodents (Healthy/Obese)	Reduced body weight gain, visceral fat and BMI. Mechanism: downregulation of lipogenic genes (PPARgamma, C/EBPalpha, SREBP-1c).	(39)
Lipid metabolism	Hypercholesterolaemic rats (Healthy/Dyslipidaemic)	Marked reduction in TC, TG and LDL-C with elevation of HDL-C. Inhibits HMG-CoA reductase and ACAT-2.	(42)
Glucose metabolism	STZ-induced diabetic mice/rats (Healthy/Diabetic)	Ameliorates hyperglycemia and improves insulin sensitivity. Protects pancreatic beta-cells and lowers HbA1c.	(39)
Hepatoprotection	Alcohol/HFD liver injury (Healthy/Metabolically stressed)	Reduces ALT/AST and prevents hepatic steatosis by increasing antioxidant enzymes (SOD, Catalase).	(43)
Antioxidant status	Various oxidative stress models (Healthy/Stressed)	Superior free-radical scavenging versus raw garlic; activates Nrf2-ARE pathway to enhance cellular antioxidant defense.	(39)
Digestive and nutritional efficiency	*Liza ramada* (Nutritionally stressed/Feed-restricted)	Elevated digestive enzyme activity (amylase, lipase, protease), improved growth and feed utilization efficiency.	(35)
Protein-energy malnutrition	Broiler—protein-restricted diet (Malnourished model)	Improved weight gain, feed conversion ratio and intestinal villus height in protein-restricted animals. Mechanistic basis: enhanced epithelial integrity and nutrient absorption—not direct caloric contribution.	(34)
Immune function	Rodent models (Various nutritional states)	Organosulfur compounds and LAB metabolites modulate immune gene expression, improve intestinal barrier integrity and enhance innate immune responses relevant to malnourished hosts.	(34, 37)

Green-highlighted rows ([NEW]) represent entries added in this revision to address malnutrition-specific evidence gaps. Zou et al. (39) and Bedrnicek et al. (12) citations corrected. HFD, high-fat diet; STZ, streptozotocin; TC, total cholesterol; TG, triglycerides; LDL-C, low-density lipoprotein cholesterol; HDL-C, high-density lipoprotein cholesterol; SOD, superoxide dismutase; PEM, protein-energy malnutrition.

Animal studies investigating the effects of fermented garlic supplementation in nutritionally stressed models have reported improvements in growth performance, nutrient utilization, and intestinal morphology. Dida et al. (34), using a broiler chicken model maintained on a protein-restricted diet (a nutritionally stressed not a healthy model relevant to PEM physiology), reported that dietary inclusion of fermented garlic was associated with enhanced weight gain, improved feed conversion efficiency, and increased intestinal villus height. These improvements most plausibly reflect indirect mechanisms enhanced digestive enzyme activity, reduced intestinal oxidative stress, and improved epithelial integrity rather than any direct macronutrient contribution by garlic itself, which is not a macronutrient source. Similarly, Moustafa et al. (35), using *Liza ramada* (a nutritionally stressed aquaculture model), demonstrated that fermented garlic increased amylase, protease and lipase activity, enhancing overall feed utilization efficiency.

Fermented garlic appears to support protein metabolism through several mechanisms: improved antioxidant capacity reduces oxidative stress associated with nutrient deficiencies, while bioactive sulfur compounds enhance metabolic efficiency and cellular protection (8, 26). Additionally, fermentation increases the concentration of bioavailable amino acid derivatives such as SAC, which may support tissue repair and growth in nutrient-deficient conditions (36). However, it must be noted that the majority of cited preclinical studies used healthy or metabolically altered (obese, diabetic, dyslipidaemic) animal models rather than models of protein-energy malnutrition (Table 1). Findings from these models cannot be directly extrapolated to malnourished humans. Well-designed mammalian PEM animal models specifically examining fermented garlic supplementation remain underrepresented in the literature and represent a high-priority research need (Section 7).

To be explicit about the asymmetry of the evidence base: the preclinical case for fermented garlic in metabolic disease contexts (hyperglycemia, obesity, dyslipidemia) is supported by multiple independent rodent studies with consistent mechanistic findings and in several cases, clear dose–response relationships. By contrast, the evidence specifically in malnutrition and PEM contexts consists of one directly relevant protein-restricted animal study [(34), broiler model] and one aquaculture nutritional efficiency study (35), neither of which used mammalian PEM models. No human clinical trial data in malnourished populations exist. This asymmetry does not invalidate the mechanistic hypothesis, it defines the primary justification for the clinical research program outlined in section 7.

### 5.2 Available human evidence: observational studies and preliminary clinical data

Available literature suggests that well-designed, controlled human clinical trials specifically examining fermented garlic supplementation in malnourished populations are currently not well established in the published literature. This constitutes a critical evidence gap and is identified as the highest-priority research need in in this review. The translational and therapeutic implications discussed therefor remain preliminary and require clinical validation before informing nutritional policy or practice.

The available human evidence relevant to fermented garlic and nutritional health can be characterized across three levels of evidence, each with important methodological limitations:

#### 5.2.1 Epidemiological and dietary data

Cross-sectional and prospective observational studies have documented associations between habitual garlic consumption and reduced markers of cardiovascular disease, oxidative stress, and chronic inflammation in population-level surveys (8). Hence, these studies typically assess raw or minimally processed garlic rather than fermented products specifically, and none have been conducted in populations defined by clinical malnutrition or EED biomarkers. Their relevance to the present review is therefore mechanistic and contextual, establishing biological plausibility rather than directly evidential.

#### 5.2.2 Preliminary clinical data on black garlic

A limited number of small-scale human studies have examined the effects of black garlic or aged garlic extract on metabolic parameters in non-malnourished adults. These studies generally report improvements in antioxidant enzyme activity, modest reductions in total cholesterol and LDL-C and decreased inflammatory markers (7, 29). However, participants in these studies are predominantly metabolically healthy or dyslipidaemic adults; no studies have examined populations with PEM, stunting, wasting, or documented micronutrient deficiencies. Dose forms and amounts vary substantially across studies, precluding meta-analysis.

#### 5.2.3 Traditional dietary evidence

Fermented garlic products have been consumed safely for centuries in Asian culinary traditions (Korea, Japan, China) without documented adverse effects at typical dietary intake levels. This provides an important safety signal supporting feasibility of dietary use, though traditional consumption patterns do not constitute evidence of therapeutic efficacy in clinical malnutrition contexts.

In summary, the available human evidence base demonstrates the biological plausibility and dietary safety of fermented garlic consumption but does not yet provide direct clinical evidence of efficacy in malnourished populations. The development of this evidence base through well-designed randomized controlled trials represents the most important research priority identified by this review.

### 5.3 Immune outcomes from preclinical models

Immune dysfunction is a major consequence of malnutrition, contributing to increased susceptibility to infectious diseases and impaired wound healing. Several preclinical studies indicate that fermented garlic may enhance immune function through multiple mechanisms, including antioxidant activity, modulation of inflammatory pathways, and gut microbiota modulation. Garlic-derived organosulfur compounds have demonstrated both antimicrobial and immunomodulatory properties, contributing to improved host defense mechanisms (37). Fermentation further amplifies these effects by generating additional bioactive metabolites and introducing beneficial microorganisms that may function as probiotics or postbiotics. Animal studies have shown that fermented garlic supplementation can influence immune-related gene expression and enhance intestinal barrier integrity, both of which are essential for protection against infection and inflammation in malnourished hosts (34).

## 6 Safety, translational potential, and limitations

All translational and therapeutic implications discussed in this review are preliminary, given that the evidence base supporting fermented garlic as a nutritional intervention in malnourished populations is predominantly derived from *in vitro* studies and animal models. Robust clinical evidence in malnourished human populations is currently underrepresented.

### 6.1 Safety considerations

Fermented garlic products are generally considered safe for human consumption and have been widely used in traditional diets across Asia and other regions for centuries without reports of serious adverse effects. The fermentation process itself improves food safety by inhibiting pathogenic microorganisms and lowering pH. Lactic acid bacteria produce antimicrobial metabolites and organic acids that contribute to product stability and microbial safety (4, 5).

#### 6.1.1 The biogenic amine safety consideration in malnourished populations

A clinical critical safety consideration specifically relevant to clinical applications in malnourished children is the biogenic amine paradox. During fermentation, certain LAB strains possessing amino acid decarboxylase enzymes can produce biogenic amines particularly histamine and tyramine through decarboxylation of histidine and tyrosine, respectively. While healthy individuals effectively catabolize dietary histamine via diamine oxidase (DAO) in the intestinal mucosa, malnourished children frequently exhibit markedly reduced DAO activity due to enterocyte atrophy and mucosal damage associated with EED (38).

This reduced DAO activity in malnourished children creates a heightened risk of histamine-mediated pseudo-allergic reactions including flushing, urticaria, gastrointestinal distress, and, in severe cases, bronchospasm upon consumption of fermented foods with elevated biogenic amine content. The clinical implication is direct and actionable: the use of well-characterized, low-amine-producing LAB starter cultures for fermented garlic production intended for vulnerable populations is an essential safety requirement. Specifically, *Lactiplantibacillus plantarum* strains confirmed to lack histidine decarboxylase (HDC) gene expression should be prioritized as starter cultures, and finished product biogenic amine content should be quantified and controlled in any clinical application. This represents a non-negotiable quality standard for therapeutic use of fermented garlic in malnourished children.

Furthermore, safety considerations include: potential interaction of garlic-derived compounds with anticoagulant medications (typically at concentrated extract doses rather than dietary intake levels); rare allergic reactions to garlic proteins (fermentation or thermal processing may reduce allergenic potential); and theoretical drug-food interactions in severely immunocompromised individuals. All of these require monitoring in clinical trial settings (8).

### 6.2 Translational potential as a complementary nutritional intervention

Fermented garlic has potential applications as a complementary nutritional intervention in two distinct modalities in LMIC settings: (1) incorporation into fortified therapeutic food formulations targeting clinical malnutrition, where its gut barrier-supportive and immunomodulatory properties may enhance the efficacy of RUTF-based protocols by addressing the EED pathophysiology that limits therapeutic food absorption; and (2) community-level integration as a fermented condiment within dietary diversification strategies for at-risk populations, leveraging its micronutrient bioavailability-enhancing properties and cultural acceptability across multiple African and Asian cuisines.

Fermented garlic products generally exhibit milder flavor profiles and enhanced palatability compared with raw garlic, they may be more acceptable for incorporation into food formulations for children and individuals with compromised appetite. Garlic is globally cultivated and remains relatively inexpensive, making fermented garlic products potentially accessible in resource-limited settings aligning with global nutrition strategies emphasizing culturally acceptable, locally available food-based interventions (2).

### 6.3 Limitations of current evidence

Despite promising experimental and mechanistic findings, several limitations restrict the direct application of fermented garlic in nutritional interventions for malnutrition. Most available evidence originates from *in vitro* studies or animal models; human clinical trials specifically examining malnutrition remain absent. Moreover, fermentation conditions vary widely across studies, differences in fermentation duration, microbial strains, temperature and processing methods can significantly influence bioactive compound profiles (18).

Furthermore, the evidence base is dominated by studies examining metabolic syndrome, dyslipidemia and obesity rather than undernutrition, PEM, wasting or stunting. Most animal models used in cited studies were healthy or metabolically stressed rather than protein-energy malnourished. Evidence directly linking fermented garlic consumption to improvements in nutritional status in malnourished humans remains critically limited, representing the primary gap in the translational pathway (32).

A structured critical appraisal of the most-cited studies further contextualizes these limitations. Zou et al. (39) the most frequently cited metabolic study, used high-fat diet rodent models with intervention periods of 4–8 weeks; while consistent outcomes were reported, relevance to undernutrition is indirect. Dida et al. (34), the most directly relevant malnutrition study, used broiler chickens, a species with gastrointestinal architecture substantially different from humans at dietary inclusion rates that are not directly translatable to human doses via standard allometric scaling. Human studies of black garlic (7, 29) involved small samples (typically *n* < 50), short durations (4–12 weeks) and enrolled metabolically healthy or mildly dyslipidaemic adults populations that differ substantially from malnourished children in LMICs in nutritional status, gut architecture, immune competence and therapeutic target.

## 7 Future research directions

Although fermented garlic has demonstrated promising physiological and biochemical properties, further research is essential to clarify its role in nutritional health and its potential for addressing malnutrition. The following key research priorities have been identified:

### 7.1 Well-designed human clinical trials in malnourished populations

This represents the highest-priority gap. Prospective randomized controlled trials are needed in populations with documented malnutrition including wasted and stunted children, populations with EED biomarkers and individuals with micronutrient deficiencies to evaluate effects of fermented garlic supplementation on nutrient absorption, intestinal morphology, immune function and recovery outcomes. Phase I dose-escalation studies informed by the allometric equivalencies provided in Table 2 below represent a logical first step.

**Table 2:** Evidence synthesis matrix of fermented garlic studies.

Study	Design/nutritional status	Fermentation conditions	Bioactive/outcome	Key finding	Quality
Zou et al (39)	Animal HFD rodent (Healthy/Obese]	Black garlic; ~ 100–200 mg/kg; 4–8 weeks	Body weight; visceral fat; lipogenic genes	Reduced weight gain and fat mass; PPARγ, SREBP-1c downregulated	P
Ryu et al (42)	Animal Hypercholesterolaemic rat (Healthy/Dyslipidaemic)	Fermented black garlic; standard protocol	TC, TG, LDL-C, HDL-C	Improved lipid profile; HMG-CoA reductase inhibited	P
Ha et al (43)	Animal HFD/alcohol liver (Healthy/Metabolic stress)	Fermented garlic extract; 8 weeks	ALT, AST; hepatic steatosis; SOD, Catalase	Reduced liver enzymes; prevented fatty liver; antioxidant enzymes restored	P
Dida et al (34)	Animal Broiler; protein-restricted (MALNOURISHED MODEL)	Dietary inclusion 0.5–1%; 35 days	Weight gain; FCR; villus height; gene expression	Improved weight gain, FCR, intestinal villus height. Most relevant to PEM.	P
Moustafa et al (35)	Animal *Liza ramada* (Feed-restricted)	0.5–2% dietary inclusion	Digestive enzyme activity; growth; immunity	Elevated amylase, lipase, protease; improved growth efficiency	P
Ryu and Kang (7) (Review)	Human Healthy/dyslipidaemic adults (NOT malnourished)	Aged black garlic; varied doses	Antioxidant capacity; lipid profile; BP	Modest antioxidant and lipid improvements; no EED/PEM outcomes	C (low)
Shin et al (29) (Sys. Rev.)	Human Mixed metabolic (NOT malnourished)	Black garlic; 4–12 weeks; *n* < 50	Antioxidant enzymes; inflammatory markers	Consistent antioxidant benefit; heterogeneous populations	C (low)
Obafemi et al (6)	Observational African populations	Traditional fermented foods; community level	Dietary quality; micronutrient status	Improved dietary quality associated with fermented food consumption	C (obs.)

Evidence Quality tiers: M, Mechanistic/in vitro; P, Preclinical animal model; C, Clinical/human. Green-highlighted row (34) is the study most directly relevant to protein-energy malnutrition. FCR, feed conversion ratio; TC, total cholesterol; TG, triglycerides; BP, blood pressure; SOD, superoxide dismutase; EED, Environmental Enteric Dysfunction; PEM, protein-energy malnutrition; obs., observational.

### 7.2 Standardization of fermentation processes

Developing standardized, reproducible fermentation protocols specifying LAB strain selection (prioritizing low-amine-producing strains), fermentation temperature, salt concentration, duration and hygiene standards would enhance study reproducibility and enable meta-analysis. This is also a prerequisite for regulatory and safety assessment in clinical applications (18).

### 7.3 Protein-energy malnutrition animal models

Studies using protein-energy malnourished rodent models (protein-restricted diets, weanling undernutrition models) examining fermented garlic supplementation effects on intestinal morphology, microbiota restoration and immune recovery are needed to strengthen the preclinical evidence base before human trials.

### 7.4 Biogenic amine control and starter culture optimization

Profiles across candidate LAB starter cultures, and validation of low-amine fermentation protocols, should be completed. LAB strains lacking HDC gene expression should be prospectively validated through both genomic screening and *in vitro* fermentation assays.

### 7.5 Gut microbiota–fermented garlic interactions in malnourished hosts

Metagenomics and metabolomics studies examining how fermented garlic modulates gut microbial composition, SCFA production and intestinal barrier biomarkers specifically in malnourished individuals would substantially advance mechanistic understanding.

### 7.6 Theoretical human equivalent doses estimates

In the current absence of human clinical dose data, theoretical human equivalent doses (HEDs) can be estimated from animal model effective doses using the FDA-recommended interspecies allometric scaling method: HED (mg/kg) = Animal dose (mg/kg) × (Animal Km ÷ Human Km), where Km correction factors are: mouse = 3, rat = 6, human = 37 (40). Table 3 presents these extrapolations for SAC and fermented garlic extract doses from cited preclinical studies. These estimates are theoretical and must be validated through prospective clinical dose-escalation trials before informing clinical practice or policy.

**Table 3:** Proposed theoretical human equivalent doses (HEDs) for fermented garlic bioactives estimated by allometric scaling from preclinical models.

Animal model	Reported dose (mg/kg BW/day)	Animal Km	Human Km	Estimated HED (mg/day, 60 kg adult)	Notes
Mouse (*Mus musculus*)	100–200 mg/kg/day SAC (39)	3	37	~16–32 mg/day	HED = Animal dose × (3/37). Preliminary estimate requiring clinical validation.
Rat (*Rattus norvegicus*)	50–100 mg/kg/day fermented garlic extract (42, 43)	6	37	~8–16 mg/day	Rat-to-human scaling. Requires prospective Phase I validation.
Broiler chicken	0.5–1% dietary inclusion (34)	N/A	N/A	Not directly scalable	Poultry allometric scaling not applicable. Dose expressed as dietary %; mechanism reference only.
Fish (*Liza ramada*)	0.5–2% dietary inclusion (35)	N/A	N/A	Not directly scalable	Aquaculture model. Mechanism data only; no scaling applicable.

FDA (40) interspecies scaling method. These are theoretical estimates only and require prospective clinical validation. N/A = not applicable (non-mammalian model; dose expressed as dietary inclusion %).

## 8 Conclusion

Fermented garlic represents a promising bioactive-dense functional food with significant potential as a complementary nutritional intervention for malnourished populations globally. Through two mechanistically distinct processing pathways which includes the microbially driven lactic acid fermentation and heat-driven thermal aging, garlic undergoes substantial biochemical transformations that improve the availability of bioactive compounds including S-allyl-L-cysteine, polyphenols and GABA. These compounds contribute to enhanced antioxidant capacity, modulation of inflammatory responses and support for gut health through prebiotic effects and SCFA production.

Critically, fermented garlic should not be positioned as a primary macronutrient source. Its most relevant potential contribution to addressing malnutrition lies in its role as a nutritional adjuvant capable of repairing the gut barrier dysfunction, mucosal immune suppression and microbial dysbiosis characteristic of Environmental Enteric Dysfunction (EED) the dominant gut pathology perpetuating malnutrition in LMICs. By addressing these underlying gut pathophysiologies, fermented garlic may improve the effectiveness of established therapeutic food programs rather than replace them.

Preclinical studies demonstrate improvements in intestinal morphology, immune responses and metabolic parameters in nutritionally stressed models, providing mechanistic proof-of-concept. However, it must be emphasized that well-designed human clinical trials in malnourished populations are absent from the current evidence base and all translational implications remain preliminary. Important safety considerations particularly the biogenic amine risk in malnourished children with reduced DAO activity must be addressed through appropriate starter culture selection and product characterization before any clinical application.

With continued scientific investigation, appropriate product standardization, evidence-based validation through controlled clinical trials and attention to safety in vulnerable populations, fermented garlic will emerge as a valuable, culturally acceptable and cost-effective complementary component of future nutrition-focused interventions aimed at improving health outcomes in malnourished populations globally. The central scientific question this review addresses whether fermented garlic can function as a gut-supportive complementary adjuvant targeting the intestinal pathophysiology of Environmental Enteric Dysfunction in malnourished populations is supported by a coherent mechanistic framework but limited by an underdeveloped clinical evidence base. The convergence of SAC-mediated Nrf2 activation, prebiotic SCFA production, NF-κB-mediated anti-inflammatory activity and sIgA-stimulating mucosal immune effects creates a mechanistically plausible foundation for EED repair. However, this framework rests almost entirely on preclinical data from non-PEM models. A coordinated research program beginning with mammalian PEM models, proceeding through Phase I dose-escalation trials using the allometric estimates in Table 3 and advancing to adequately powered RCTs with EED biomarker endpoints is required before fermented garlic can be responsibly recommended as a clinical tool. This review provides the mechanistic foundation and research roadmap for that program.

## References

[1] UNICEF. The state of the World's children 2023: For every child, Nutrition. New York, NY, USA: UNICEF (2023).

[2] WHO. Global Nutrition Report 2022. Geneva, Switzerland: World Health Organization (2022).

[3] Humphrey JH. Child undernutrition, tropical enteropathy, toilets, and handwashing. Lancet. (2009) 374:1032–5. doi: 10.1016/S0140-6736(09)60950-8, 19766883

[4] Tamang JP Cotter PD Endo A Han NS Kort R Liu SQ . Fermented foods in a global age. Compr Rev Food Sci Food Saf. (2020) 19:184–217. doi: 10.1111/1541-4337.12520, 33319517

[5] MarcoML SandersME GänzleM ArrietaMC CotterPD De VuystL . ISAPP consensus statement on fermented foods. Nat Rev Gastroenterol Hepatol. (2021) 18:196–208. doi: 10.1038/s41575-020-00390-5, 33398112 PMC7925329

[6] ObafemiYD OranusiSU AjanakuKO AkindutiPA LeechJ CotterPD. African fermented foods: overview, emerging benefits, and novel approaches to microbiome profiling. npj Sci Food. (2022) 6:15. doi: 10.1038/s41538-022-00130-w, 35181677 PMC8857253

[7] RyuJH KangD. Physicochemical properties and health benefits of aged black garlic. Molecules. (2017) 22:919. doi: 10.3390/molecules2206091928587168 PMC6152780

[8] ShangA CaoSY XuXY GanRY TangGY CorkeH . Bioactive compounds and biological functions of garlic (*Allium sativum* L.). Foods. (2019) 8:246. doi: 10.3390/foods8070246, 31284512 PMC6678835

[9] Javed H Ahamed M. Fermented garlic: processing methods and bioactive compound development. Food Chem Rev. (2022) 8:44–58.

[10] Hing K. Lactic acid fermentation in vegetable preservation: an overview. Int J Food Ferment Technol. (2020) 11:125–42.

[11] Suresh M. Lactic acid production and food safety in fermented vegetables. Journal of Applied Food Science. (2022) 15:201–18.

[12] BedrnicekJ SalekRN CernaM TremlovaB. Bioaccessibility and content of bioactive compounds in black garlic after in vitro digestion. Foods. (2021) 10:730. doi: 10.3390/foods1004073033808212 PMC8065589

[13] Moreno-OrtegaA García-RuizA SampedroI. Maillard reaction products in aged black garlic: formation, characterisation, and bioactivity. Foods. (2023) 12:2876. doi: 10.3390/foods1215287637569147 PMC10417461

[14] SrinivasP KamilS PalS SharmaP. Metagenomic analysis of lactic acid bacteria in fermented vegetable products. Appl Microbiol Biotechnol. (2022) 106:3231–45. doi: 10.1007/s00253-022-11884-835416487

[15] Zheng J Wittouck S Salvetti E Franz CM Harris HM Mattarelli P . A taxonomic note on the genus Lactobacillus: description of 23 novel genera. Int J Syst Evol Microbiol. (2020) 70:2782–858. doi: 10.1099/ijsem.0.004107, 32293557

[16] KimY LeeJ ParkH. Metabolic adaptation of lactic acid bacteria in acidic fermented food environments. Food Microbiol. (2021) 95:103691. doi: 10.1016/j.fm.2021.10369133397620

[17] LeeSJ KangMJ ChoiDJ SungNJ. Bioactive compounds in lactic acid fermented garlic and their health-promoting properties. Molecules. (2022) 27:2431. doi: 10.3390/molecules2708243135458629 PMC9033138

[18] XuY FengY. Biological activities and fermentation mechanisms of garlic products. Food Chem. (2020) 321:126689. doi: 10.1016/j.foodchem.2020.12668932259732

[19] Nam YD Park SH Lim SI. Microbial succession dynamics in garlic fermentation revealed by high-throughput sequencing. Front Microbiol. (2023) 14:1098742. doi: 10.3389/fmicb.2023.1098742

[20] Mathur H Begley M Cotter PD Hill C Ross RP. Bacteriocins in vegetable fermentations: a review. J Food Prot. (2020) 83:548–60. doi: 10.4315/0362-028X.JFP-19-348

[21] da Silva FernandesM EscaramboniB de Oliva NetoP. Yeast diversity in fermented foods and beverages: a review. Int J Food Microbiol. (2022) 378:109821. doi: 10.1016/j.ijfoodmicro.2022.10982135816956

[22] Alharbi SA. Synergistic effects of yeast-LAB co-fermentation on metabolite diversity and antioxidant potential in fermented vegetables. J Appl Microbiol. (2026) 140:e16785. doi: 10.1093/jambio/lxad143

[23] Singh P Kumar R. Environmental determinants of LAB community structure in brine-fermented vegetables. LWT-Food Sci Technol. (2025) 192:115072. doi: 10.1016/j.lwt.2025.115072

[24] Fadare O Akintonwa A Oladimeji F Adebayo-Tayo BC. Bioactive compounds and antimicrobial properties of fermented garlic (*Allium sativum* L.). J Food Biochem. (2022) 46:e13987. doi: 10.1002/jfbc.13987

[25] Li X Zhang Z Chen W. Enzymatic conversion of organosulfur compounds during garlic fermentation: a mechanistic review. Food Chem. (2023) 405:134712. doi: 10.1016/j.foodchem.2022.134712

[26] Kim JH Nam SH Rico CW Kang MY. A comparative study on the antioxidative and anti-diabetic activities of fermented and non-fermented black garlic extracts. Food Chem Toxicol. (2018) 114:347–54. doi: 10.1016/j.fct.2018.01.050

[27] Hanley SK. Exopolysaccharide production by lactic acid bacteria in vegetable fermentations. Int Dairy J. (2025) 148:105792. doi: 10.1016/j.idairyj.2025.105792

[28] Shah NP Shim YY. Fermented foods: production, characterisation, and health benefits. Curr Opin Biotechnol. (2025) 91:102847. doi: 10.1016/j.copbio.2025.102847

[29] Shin JH Kim SH Kang MJ Hwang CR. Health-promoting effects of black garlic: a systematic review. Food Rev Int. (2022) 38:614–32. doi: 10.1080/87559129.2020.1771503

[30] Zhao Y Sun Y Gao Y Li M Wu X. Fermentation characteristics and functional properties of black garlic. Food Biosci. (2022) 45:101501. doi: 10.1016/j.fbio.2022.101501

[31] Ríos-Covián D Ruas-Madiedo P Margolles A Gueimonde M de Los Reyes-Gavilán CG Salazar N. Intestinal short-chain fatty acids and their link with diet and human health. Front Microbiol. (2020) 11:566876. doi: 10.3389/fmicb.2020.566876 PMC4756104 26925050

[32] Zhang H Zhang M Liu Y Liu R Wang Y. Probiotic characteristics of *Lactiplantibacillus plantarum* isolated from fermented foods. J Appl Microbiol. (2019) 127:1645–57. doi: 10.1111/jam.14390

[33] Monteagudo E Martín-Peláez S López-Sabater C Ferrer E. Bioactive compounds and antioxidant capacity of black garlic. Eur Food Res Technol. (2019) 245:195–202. doi: 10.1007/s00217-019-03364-x

[34] DidaMM SrithongchaiW ThongsomS NithikulworawongN RungsiwiwutR. Influence of fermented garlic on growth performance, intestinal morphology, and gene expression in broiler chickens. Anim Biosci. (2021) 34:1903–12. doi: 10.5713/ab.21.005034237926

[35] Moustafa AAA Hassan MA Elgendy MY. Exploring the dual benefits of fermented and non-fermented garlic as dietary supplements: impacts on growth, immunity, and antioxidant status in *Liza ramada*. Fishes. (2024) 9:389. doi: 10.3390/fishes9100389

[36] Kodera Y Ushijima M Amano H Suzuki J. Chemical and biological properties of aged garlic extract. J Nutr. (2017) 147:867–74. doi: 10.3945/jn.116.236208 PMC6154623 28362335

[37] BayanL KoulivandPH GorjiA. Garlic: a review of potential therapeutic effects. Avicenna J Phytomed. (2014) 4:1–14. doi: 10.22038/AJP.2014.174125050296 PMC4103721

[38] EFSA Panel on Biological Hazards. Scientific opinion on the risks associated with biogenic amines in fermented foods. EFSA J. (2023) 21:e07891. doi: 10.2903/j.efsa.2023.e07891

[39] Zou Y Wang L He Z Li Q Jiang G. Black garlic attenuates obesity by reducing fat accumulation in high-fat diet-induced obese rats. J Funct Foods. (2019) 54:487–95. doi: 10.1016/j.jff.2019.03.043

[40] FDA. Guidance for Industry: Estimating the Maximum Safe Starting Dose in Initial Clinical Trials for Therapeutics in Adult Healthy Volunteers. Rockville, MD, USA: U.S. Department of Health and Human Services (2005).

[41] RaisN AhmadM MuddassirM AhmadFJ. S-allyl-L-cysteine: physicochemical nature, mechanism, pharmacokinetics, and health-promoting activities. Molecules. (2023) 28:1148. doi: 10.3390/molecules2803114836770815 PMC9920184

[42] Ryu JH Kim JY Kang D. Hypolipidaemic effects of fermented black garlic in hypercholesterolaemic rats. J Funct Foods. (2019) 56:74–82. doi: 10.1016/j.jff.2019.01.040

[43] Ha AW Kim JH Kho Y. Comparison of efficacy of fermented garlic and orlistat in high-fat diet-induced obese rats. J Med Food. (2023) 26, 747–754. doi: 10.1089/jmf.2023.K.0049

[44] Bihan DL Ha V Cotter PD Walsh AM. Fermentation of plant-based proteins: microbiology, chemistry, and applications. Trends Food Sci Technol. (2022) 120:210–25. doi: 10.1016/j.tifs.2022.04.021

[45] González-Alonso V Jiménez-Valera M Ruiz-Bravo A Beltrán D. Lacto-fermented garlic handcrafted in the lower Silesia region (Poland): microbial diversity, morpho-textural traits, and volatile compounds. Food Res Int. (2024) 175:113770. doi: 10.1016/j.foodres.2024.113770 38823870

[46] Mastella L Polo A Gianotti A. Yeast contributions to flavour complexity in plant-based fermented beverages. LWT. (2023) 175:114482. doi: 10.1016/j.lwt.2023.114482

[47] Pentrova D Vlček J Horáček J. Early fermentation stage LAB ecology and their role in bioactive metabolite formation. Eur J Food Res Technol. (2022) 248:1041–52. doi: 10.1007/s00217-021-03920-2

[48] ZikmanisP MezuleL LiepinsJ. Early-stage LAB succession in brine vegetable fermentations: ecological and metabolic determinants. FEMS Microbiol Lett. (2020) 367:fnaa147. doi: 10.1093/femsle/fnaa14732860684

