# Supplementary material for: Fermented garlic as a functional food strategy for malnutrition: microbial ecology, bioactive compounds, and clinical perspectives
Source: Front Nutr. 2026 Jun 11;13:1839155. doi: 10.3389/fnut.2026.1839155 (PMC13294105; doi:10.3389/fnut.2026.1839155)
Supplement: Supplementary file 1 [file Supplementary_file_1.pdf]

SUPPLEMENTARY FIGURE S1

PRISMA 2020 Flow Diagram – Literature Search and Study Selection

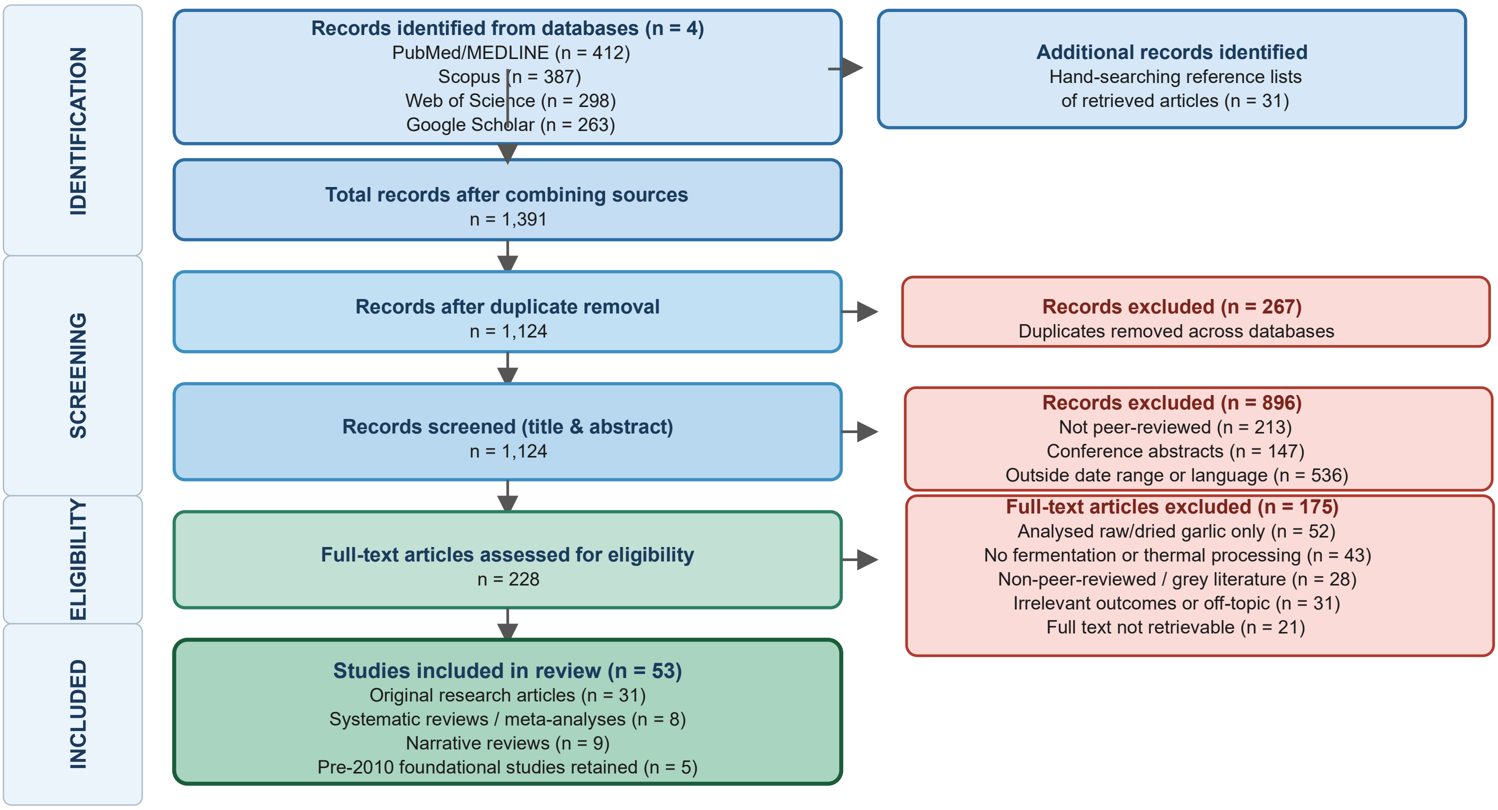

Notes:

Search conducted: January 2010 – March 2026. Databases: PubMed/MEDLINE, Scopus, Web of Science, Google Scholar.
Searches conducted independently by two authors (MAE-F and YDO); discrepancies resolved by consensus with supervising author (SUO). Pre-2010 foundational studies (n = 5) included where mechanistically essential, per inclusion criteria stated in Section 1.1.
Prepared in et al. (2021) BMJ 372:n71. doi:10.1136/bmj.n71
